# The Effect of Sweet Taste on Romantic Semantic Processing: An ERP Study

**DOI:** 10.3389/fpsyg.2019.01573

**Published:** 2019-07-09

**Authors:** Liusheng Wang, Qian Chen, Yan Chen, Ruitao Zhong

**Affiliations:** ^1^Department of Psychology, Nantong University, Nantong, China; ^2^School of Psychology, Jiangxi Normal University, Nanchang, China; ^3^Fuzhou Medical College, Nanchang University, Fuzhou, China

**Keywords:** embodied cognition, romantic, sweet taste, semantic processing, knowledge activation, cross-modal processing, semantic richness

## Abstract

Previous studies have found that sweet perception affects the subjective evaluation of interpersonal intimacy and romantic semantic processing. However, the cognitive processes involved in this effect are unclear. The aim of the current study was to investigate the sweet-love embodied effect in semantic processing and its underlying mechanism by Event-Related potentials technique. Participants were randomly exposed to sweet-taste or tasteless conditions, during which they performed a lexical decision-task that involved romantic and non-romantic words. The results showed an enhanced N400 for romantic words compared to non-romantic words in the sweet-taste condition, and a larger P200 for romantic words relative to non-romantic words. The results demonstrate that taste sensations can cross-modally facilitate the semantic processing of romance. These findings support the embodied effect of sweet-love and are discussed from the perspective of embodied cognition with knowledge activation of concept and semantic richness.

## Introduction

Some studies have found that the perception of sweet taste and psychological feelings of love are interconnected and interact with each other. Chan et al. (2013) found that being in a romantic mental state can affect the perception of a sweet taste; namely, love promotes the perception of a sweet taste. Ren et al. (2014) examined the effect of sweetness on love, and found that a sweet taste can enhance the positive evaluation of potential dating objects and a subjective romantic attitude. Further, Wang and Chen (2018) found that experiencing sweet taste can affect romantic semantic processing, and has the romantic advantage effect that participants in a sweet taste condition process romantic words more rapidly than processing non-romantic-words. Romantic semantic priming also has the enhancement effect of sweetness (Li and Wang, 2018). With regard to semantic processing, there exists a cross-modal processing of flavor perception and linguistic information (words), showing a facilitation effect (Razumiejczyk et al., 2015, 2017), or taste-shape correspondences (Velasco et al., 2018); for instance, the “sweet and sour” food concept is associated with pairs of round and/or angular shapes.

Cross-modal processing, or cross-modal integration and cross-modal interference, refers to the processing in which the stimulation of, or experience in, one sensory modality influences the processing of stimuli presented in a different modality (Spence and Deroy, 2013; Razumiejczyk et al., 2015, 2017). It happens not only in the perception processing from one modality to another (Spence et al., 2001), such as a light in vision, a touch on a finger, a tone in audition, but also in concept or semantic processing involving a shift within perceptional modalities (Pecher et al., 2003): occurring in visual, auditory, gustatory, or tactile fields, e.g., BLENDER—loud, vs. CRANBERRIES—tart; shifts between perceptual and conceptual fields (Van Dantzig et al., 2008); and a switch from the affective system to sensory modalities, and vice-versa (Vermeulen et al., 2007; Tillman et al., 2013; Wang and Li, 2014), e.g., APPLE–red vs. MOTHER–respected. Following the embodied cognition, cross-modal processing involving the knowledge of concepts or semantic processing, holds that perceptual simulation underlies conceptual processing, perceptual processing can affect the activation of conceptual knowledge, and perceptual and conceptual representations are at least partially based on the same systems (Simmons et al., 2007; Barsalou, 2008; Wang and Li, 2014; Razumiejczyk et al., 2015; Razumiejczyk et al., 2017).

Embodied cognition emphasizes the role of the body in cognition and holds that physical experiences influence psychological processing (Barsalou, 2003, 2008). A large number of empirical studies have found embodied effects. For example, body warmth can evoke interpersonal warmth (Williams and Bargh, 2008), the concept of mighty is linked to the vertical dimension (Zanolie et al., 2012), and the perception of interpersonal spatial distance affects interpersonal psychological distance (Wang and Yao, 2016), and olfactory cues can elicit social suspicion (Lee and Schwarz, 2012). As has been shown by some studies on the embodied effect, it is possible that some factors would affect the manifestation of the effect (Dong et al., 2010; Denke et al., 2016). For instance, psychological distance modulates the “good-pulling”/“bad-pushing” action-evaluation embodiment effect (Wang et al., 2015). A study by Chandler et al. (2012) on the concept of importance found that the perceived weight of a book affects evaluation of the importance of the book, but the effect only occurs when subjects had a substantial understanding of the book.

As far as concept or semantic processing is concerned, it is involved in knowledge activation and semantic richness. Semantic richness is the semantic information generated by people considering the meaning of concepts, including the number of semantic features (NF), semantic neighborhood density (SND), the number of distinct first associates (NoA), imageability, the number of senses (NS), body-object interaction (BOI), and so on (Pexman et al., 2007; Yap et al., 2012). Higgins (1996) proposed that knowledge activation of a concept involves three stages, or so called principles, including availability, accessibility, and applicability (Todorov, 2000; Förster and Liberman, 2007; Wyer, 2008). Availability refers to whether or not some particular knowledge is actually stored in memory. It is a necessary condition for accessibility. Accessibility can be defined as the activation potential of available knowledge, and exposure to information related to a construct leads to the higher accessibility of the construct and increases the likelihood of its use (Todorov, 2000). Some studies evidenced that the concept with richer semantic representation is much easier to be accessed, such as concrete words over abstract words (Rabovsky et al., 2012; Amsel and Cree, 2013), to build stronger attractors in semantic space and to allow more efficient semantic processing (Pexman et al., 2007). Applicability refers to the relation between the features of some stored knowledge and the attended features of a stimulus. For example, physical movements can elicit proprioceptive feedback. This internally generated stimulation can serve as features of a cognitive production that, in combination with other stimulus features, spontaneously elicit a sequence of behavior (Wyer, 2008).

The accessibility and applicability of a concept's knowledge may be a requirement for the embodied effect of concept processing (Landau et al., 2011; Lee and Schwarz, 2012). Sensorimotor experience, e.g., fishy smells, affects the associated psychological experience like suspicion, only when knowledge of concept is available to the person, accessible in the context, and applicable to the target (Lee and Schwarz, 2012), namely, embodied effect may depend on the activation of concept's knowledge, such as knowledge's accessibility and applicability. Once accessible, knowledge of a concept can affect an individual's perceptions of, feelings about, and behavior toward an applicable target.

Although embodied effects involving the perception of sweet taste and romantic semantics, are found to be bidirectional (Li and Wang, 2018; Wang and Chen, 2018), and are explained from embodied cognition and knowledge activation of the concept, the mechanism of the process still remains unclear. The arguments made are mainly constructed by the behavioral index, such as reaction time, or subjective rating, which are not adequate to reveal the embodied effect in semantic processing from the perspective of knowledge activation. Event-related potentials (ERPs) allow researchers to closely investigate the time course of processing with high temporal resolution. If romantic semantics are processed after one's experiencing sensation and perception, it should involve the component P200 and N400 in ERPs study, which may make it possible to understand the sweet-love effect at the semantic level more clearly. Previous studies have shown that emotional valence words have an enhanced P200 amplitude (Bernat et al., 2001; Kanske and Kotz, 2007), and semantic richness is associated with reaction times (Pexman et al., 2008; Grondin et al., 2009), P200, and N400 component (Lau et al., 2008; Kounios et al., 2009; Kwon et al., 2012; Rabovsky et al., 2012; Xue et al., 2017), and can also be influenced by context (Schwanenflugel and Shoben, 1983; Levy-Drori and Henik, 2006; Tousignant and Pexman, 2012) or different measures of semantic richness (Rabovsky et al., 2012; Yap et al., 2012). P200 is a component for emotional information processing (Bernat et al., 2001; Kanske and Kotz, 2007). Romantic words have richer semantic information over non-romantic words. If romantic words activate the P200, it means that romantic knowledge of words is available, which could be accessible under some conditions. Recent studies also support that semantic richness effects are associated with the N400 effect (Rabovsky et al., 2012; Tousignant and Pexman, 2012; Yap et al., 2012; Amsel and Cree, 2013). Hence, the N400 effect can be used as an indicator of knowledge accessibility and applicability. According to the knowledge activation of concept and embodied theory, if the embodied effect of sweet-love does exist, romantic advantage effect can be divided into different taste conditions. There is a romantic advantage effect in the sweet-taste group, but not in the tasteless group. If the romantic words would lead to more efficient processing, romantic semantics would be the available knowledge, which is more easily accessible in the sweet-taste condition, and more applicable in a sweet-taste context. Therefore, the current study used the ERPs technique to examine the sweet-love effect in the semantic processing through a 2 (taste: sweet, tasteless) × 2 (romance: romantic word, non-romantic word) mixed design.

## Materials and Methods

### Participants

A total of 28 college students were paid to participate in the experiment, however, data from only 23 students were included in the statistical analysis; two students did not correctly judge sweetness, one student's electrode impedance was above 5 kΩ, one student guessed the purpose of the experiment, and one student's EEG signals had serious drift during recording. Twelve participants were male and 11 were female; *M* = 21.39 years of age, *SD* = 2.08. Participants were right-handed, had normal vision or corrected visual acuity, and had no self-reported history of physiological or mental illness.

### Materials

#### Vocabulary

The “*Chinese Affective Words System*” (CAWS) (Wang et al., 2008) and the “*Modern Chinese Dictionary* (7th Edition)” were used to select 140 positive words. We used the “*Modern Chinese Word Frequency Dictionary*” (1986) to check the word frequency. Sixty-one college students were randomly selected to assess the valence, concreteness, familiarity, and arousal of the vocabulary words on a 9-point scale (for example, 1 = Not at all familiar; 5 = Ordinary; 9 = Extremely familiar). There was a total of 48 positive words, 24 romantic words (8 nouns, 8 adjectives, and 8 verbs), and 24 non-romantic words (8 nouns, 8 adjectives, and 8 verbs) (see the Appendix). Fifty college students were randomly selected to assess the degree of romance expressed by the words. In addition, there were 48 pseudo words (for example: 门躺, door-lie; 海矮sea-short) that were used as filler trials designed to mask the purpose of the experiment.

Statistical analyses showed that the romantic words and the non-romantic words were significantly different in terms of their degree of romance, *t*_(46)_ = 16.464, *p* < 0.001, but did not differ significantly in terms of their valence, arousal, concreteness, familiarity, or word frequency, *p*s > 0.05 (see Table 1). The mean valence of the words was close to 7, indicating a positive valence for all the words.

**Table 1:** Mean ratings of the characteristics of romantic and non-romantic words.

	**Romantic word**		**Non-romantic word**		***t***
	***M***	***SD***	***M***	***SD***	
Valence	6.92	0.32	6.85	0.40	0.675
Concreteness	5.54	0.72	5.40	0.53	0.782
Romance	6.94	0.76	3.27	0.78	16.464^***^
Familiarity	6.62	0.11	6.64	0.21	−0.442
Arousal	6.14	0.37	5.97	0.35	1.655
Word Frequency	0.009	0.0389	0.009	0.0039	0.000

*** *p < 0.001*.

### Experimental Intervention

Rock candy was used as the sweetener in the sweet-taste condition; white kidney beans were used in the control condition. In the formal experiment, the participants were asked to rinse their mouths with water and to keep the experimental material above the central part of the tongue. The position of the experimental material could not be moved during the experiment, but the participants could swallow normally to ensure that they had a taste experience.

### Procedure

The basic principles of ERP recording were explained to the participants before they gave their signed informed consent. Participants wore an electrode cap and sat about 80 cm from the front of the computer screen, keeping their head fixed. Before the experiment began, participants were informed that they had to focus on the “+” at the center of the screen during the entire experiment, and to try to avoid blinking. They performed the lexical decision task in which they decided if the word was a real or a pseudo word, with the experimental material in their mouths. There was a break for rest through the experiment. The experiment was programmed in E-prime software. Participants were given practice trials before the formal experiment started. The participants rated the taste stimulus on a 10-point scale (0 = Tasteless; 9 = Extremely sweet) when they completed the experiment.

**Figure 1:**
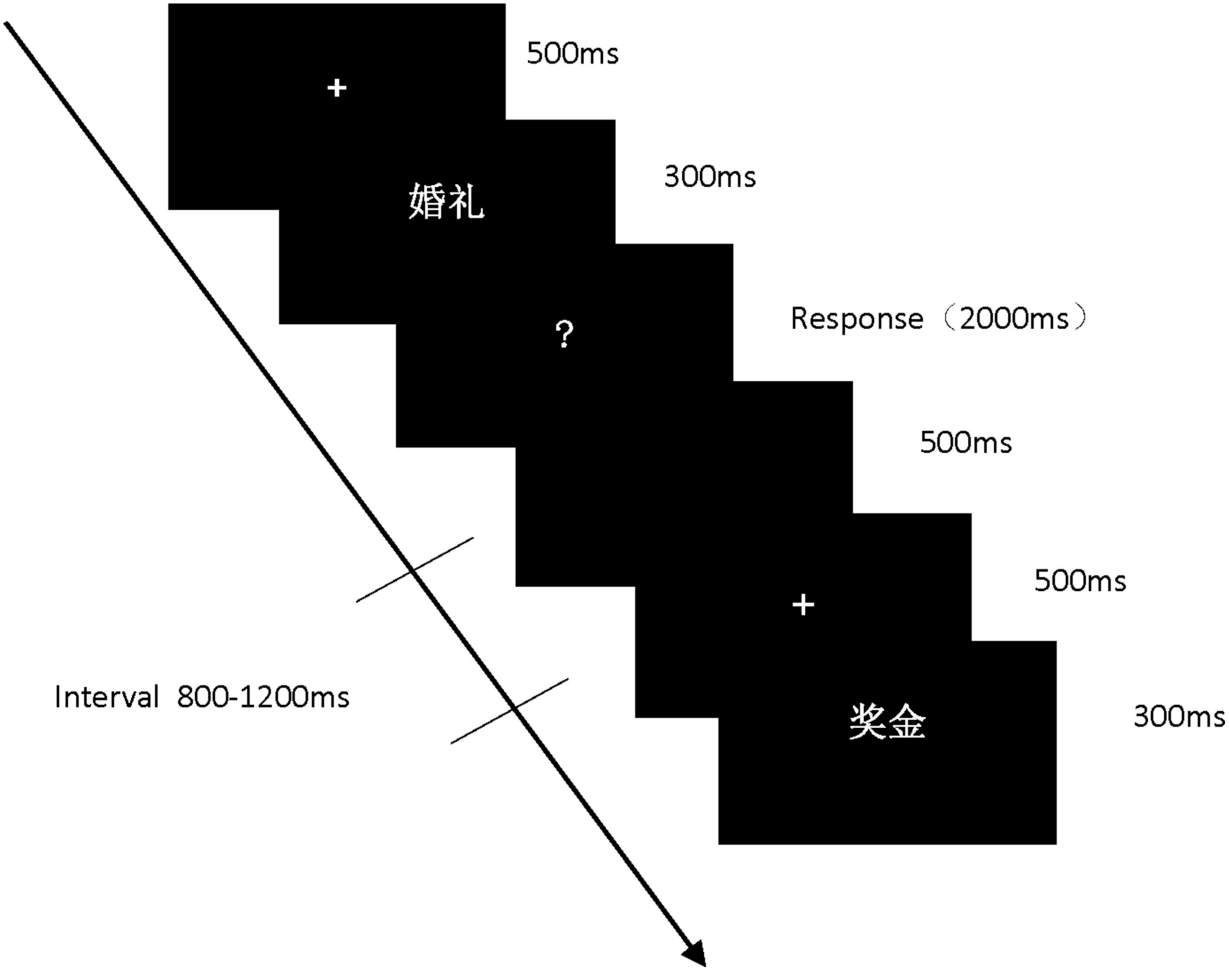
The sequence of experiment.

All the words were presented at random during the experiment. The experimental procedure is shown in Figure 1. First, the fixation point (“+”) was presented at the center of the computer screen for 500 ms, then words appeared for 300 ms, followed by a “?” interface; when the “?” interface was presented, participants were required to make a judgment whether the preceding words were real or not. They were instructed to use their left (right) hand index finger to press the F key if the word was real, and to use their right (left) hand index finger to press the J key if it were a pseudo word, balanced for participants' use of their left and right hands. The word disappeared if no response was made within 2,000 ms, and a blank screen appeared for 500 ms. The fixation point appeared after the end of each trial for a random interval of 800 to 1,200 ms, before the next trial began. The computer screen had a black background, and the words were presented in white 48 Song typeface. The entire experiment took about 25 min to complete.

### ERP Recording and Analysis

The experiment was conducted in a standard Electroencephalogram (EEG) laboratory. EEG signals were recorded using a 64 Ag-AgCl electrode elastic-cap placed according to the international 10–20 system, using the Neuroscan ERP workstation (scan 4.5). The reference electrode was on the left mastoid (M1) and the ground electrode (GND) was on the medial frontal aspect. A vertical electrooculogram (EOG) was recorded from above and below the participant's left eye, and a horizontal EOG was recorded from the outer canthi of both eyes. All electrode impedance was kept below 5 KΩ. The sampling rate and band pass were 1,000 Hz/channel and 0.05–100 Hz. All participants washed their hair in the laboratory, and recording did not start until the impedance was stable (below 5 KΩ) with the conductive paste on the scalp.

The EEG data were processed offline using the Neuroscan ERP workstation (scan 4.5). First, we fused the EEG data with the behavioral data, and the average of M1 and M2 was converted to a new reference. Ocular artifacts (mean EOG voltage exceeding ±100 μV) related to blinks and vertical eye movements were removed from the EEG by ocular correction. According to the attributes of the stimulus and trials with correct response segmentation, the analysis epoch was from 200 ms before baseline to 1,000 ms after stimulation. After baseline correction, trials with excessive artifacts (±80 μV) were removed from further analysis. The EEG wave forms for correct responses in each condition were overlapped and averaged separately. The EEG analyses were performed on the differences in mean wave forms for all types of conditions.

According to the grand average map and previous research (West and Holcomb, 2006; Wang et al., 2019), the ERP components were divided by the time windows in which they occurred, at time window 200–300 ms (P200), and at 400–520 ms (N400). Based on the grand average map and previous research (Bartels and Zeki, 2000; Wang et al., 2015; Luo et al., 2018), we selected representative 12 recording electrodes as our region of interest (FP1/2, FPZ; F3/4, FZ; C3/4, CZ; P3/4, PZ). The *p*-value was corrected by the Greenhouse-Geisser method for repeated measures analysis of variance.

## Results

After removing invalid data from five participants, the average rating for sweetness for sweet-taste condition was 6.18 (0 = Tasteless, 9 = Extremely sweet). It was agreed that the control condition was tasteless. A repeated-measures ANOVA was conducted on the mean amplitudes of the P200 and N400 components, using electrode site, taste, and romance as factors.

The results for the P200 amplitude showed a significant main effect of romance, *F*_(1, 21)_ = 7.593, *p* < 0.05, η_*p*_^2^ = 0.266, with romantic words (7.023 μv) inducing a larger P200 compared to non-romantic words (6.417 μv). The main effect of taste was also significant, *F*_(1, 21)_ = 5.566, *p* < 0.05, η_*p*_^2^ = 0.210; words in the tasteless condition (8.108 μv) induced a stronger P200 than words in the sweet-taste condition (5.331 μv). The main effect of the electrode site also was significant, *F*_(11, 231)_ = 30.010, *p* < 0.001, η_*p*_^2^ = 0.588. There was no significant interaction between romance and taste, *F*_(1, 22)_ = 0.783, *p* = 0.39.

**Figure 2:**
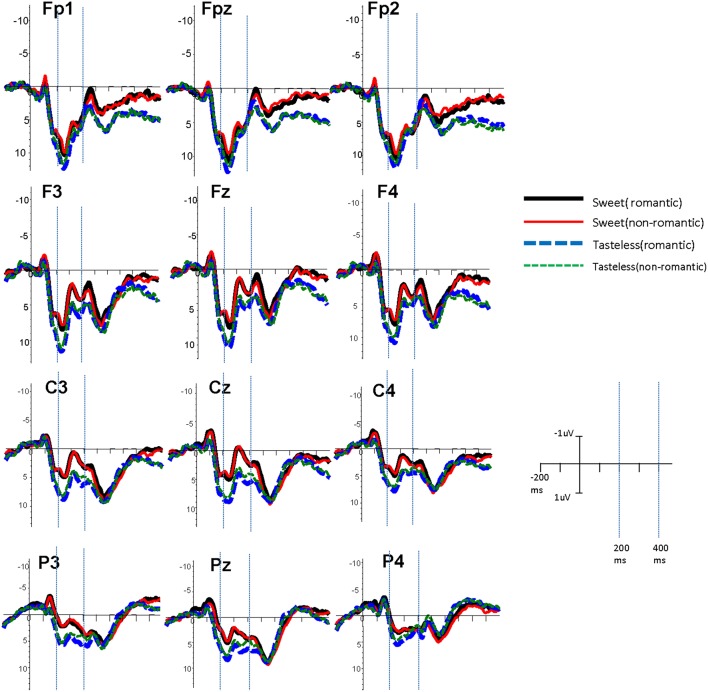
Grand average waveforms showing the romantic and non-romantic words processed in different taste conditions.

An ANOVA on the N400 amplitude revealed no significant main effect of romance, *F*_(1, 21)_ = 0.662, *p* = 0.43, or taste, *F*_(1, 21)_ = 0.937, *p* = 0.34. However, the main effect of electrode site was significant, *F*_(11, 231)_ = 4.485, *p* < 0.05, η_*p*_^2^ = 0.176. The interaction between romance and taste was also significant (see Figure 2), *F*_(1, 21)_ = 5.017, *p* < 0.05, η_*p*_^2^ = 0.193. Furthermore, a simple effect analysis showed that the N400 amplitude was more negative-going for romantic words (3.096 μv) than for non-romantic words (3.749 μv) for participants in the sweet-taste condition, *F*_(1, 21)_ = 4.47, *p* < 0.05. No significant difference was found in the tasteless condition, between romantic words (4.891 μv) and non-romantic words (4.891 μv), *F*_(1, 21)_ = 1.06, *p* = 0.31.

## Discussion

The aim of this study was to examine the sweet-love effect in semantic processing using the ERPs technique, and to analyze the embodied effect under the framework of knowledge activation of concept. The experimental results showed a larger P200 and enhanced N400, indicating a romantic advantage effect. That is to say, sweet taste sense can promote the processing of romantic words, which is in line with the conclusions of behavior experiments (Ren et al., 2014; Wang and Chen, 2018).

The current study found that romantic words induced a larger P200 amplitude than non-romantic words did. The P200 is an index for emotional information processing (Bernat et al., 2001; Kanske and Kotz, 2007). Therefore, the enhanced P200 indicated the availability of the romantic component in the knowledge of the concept. More importantly, the study found an enhanced N400 for romantic words compared with non-romantic words in the sweet-taste condition, and no difference existed between romantic words and non-romantic words in the control condition. The promoting effect of sweet taste on romance is present in semantic processing, indicating a romantic advantage effect. Richer semantic information for romantic words induced an enhanced N400. Previous studies found semantic richness has contradictory findings from event-related potential, demonstrating an enhanced N400 or an attenuated N400 (Rabovsky et al., 2012; Amsel and Cree, 2013). The semantic richness effect may connect with context (Levy-Drori and Henik, 2006; Amsel and Cree, 2013), or different measures of semantic richness (Rabovsky et al., 2012; Yap et al., 2012). In this study, the experimental task (lexical decision task) and semantic richness (romance) are similar to the study by Rabovsky et al. (2012), showing an enhanced N400. These results are also consistent with the results of previous behavioral studies about the effect of sweet taste sense on romantic words processing (Wang and Chen, 2018). Based on the results of ERPs in the current study and behavioral results of previous studies (Chan et al., 2013; Ren et al., 2014; Li and Wang, 2018; Wang and Chen, 2018) the embodied effect of sweet-love indeed exists, and the effects are bidirectional.

There is an alternative explanation for the embodied effect of sweet-love in semantic processing from the perspective of knowledge activation of concept. Sweet taste appears to make a romantic word more accessible at the time, and distinguishes it from non-romantic words in the N400 effect, illustrating the cross-modal processing effect of sweetness involving taste sense and semantics. The ERPs results further support the arguments of Lee and Schwarz (2012) that sensorimotor experiences should affect embodied associated psychological experiences, only when knowledge is available to the person, accessible in the context, and applicable to the target. It is similar to the facilitation effect of flavor on language (Razumiejczyk et al., 2015, 2017).

The P200 reflects the processing romantic information in this study, that is, romantic information of semantic words is available during the entire process. Sweet taste has romantic semantics accessible and distinguishes them from a “tasteless” taste. Finally, after a sweet cue is accessible and applied to a specific goal (in this study, the romantic words) romantic words are distinguished from non-romantic words, which is present in N400 effect. Combined with results of an earlier study (Ren et al., 2014), it is concluded that sweet taste affects romantic concepts processing as well as embodied behavior. On the other hand, when relevant knowledge is not available, or not directed to a target, there is no necessity for an embodied effect to occur (Lee and Schwarz, 2011, 2014). The embodied effect of sweet-love cannot occur unless the related knowledge, such as romance in semantic words, is accessible and can be applied to the target (Chandler et al., 2012; Lee and Schwarz, 2012).

In general, the sensation of the body functions a clue to cognition and increases the accessibility of knowledge related to embodied effect. Sensorimotor experience sets the direction of cognition based on embodied cognition framework (Lee and Schwarz, 2010; Denke et al., 2016). In this experiment, sweet taste is a clue that allows participants to cross the modality to romantic semantics. In light of the above, it is vital for embodied effect that body clues combine with current environmental clues, leading to improving the accessibility of knowledge of concept. A person's cognition is subject to the physical properties of the body and the activation of the person's knowledge. From an evolutionary perspective, individuals are always seeking the most appropriate way to survive and develop. As physical experience is a weak clue, the effect of this experience is subject to a large number of internal and external factors, while individuals in different situations start from their own perspective to make the best choice. For instance, studies have shown that the weight of an object influences the judgment of importance (Ackerman et al., 2010; Chandler et al., 2012), but elaborative thinking can further influence this effect (Hauser and Schwarz, 2015). Or, people can achieve cognitive goals through a number of ways in combination with the current environment, but people eventually choose the most suitable way to do so. The embodiment effect is similar; when the body clues are present and the current situation is in place, the embodied effect pops out to promote the efficiency of the individual's cognition. Therefore, internal and external factors should be considered for psychological processing.

Overall, the study found a romantic advantage effect by an enhanced N400, indicating that sweet sense can improve semantic processing of romance. This cross-modal embodied effect involves knowledge activation of concept, supporting the notion that the mind is rooted in the cross-modal interaction of the body and the environment.

## Ethics Statement

This study was carried out in accordance with the recommendations of Nantong University Committee with written informed consent from all subjects. All subjects gave written informed consent in accordance with the Declaration of Helsinki. The protocol was approved by Nantong University Committee.

## Author Contributions

LW developed the study concept. LW and QC contributed to the design, implemented the experiment, collected and analyzed the data, wrote and revised the manuscript. YC participated in the analysis of ERP data. RZ gave some advice involving the understanding of neuroscience.

### Conflict of Interest Statement

The authors declare that the research was conducted in the absence of any commercial or financial relationships that could be construed as a potential conflict of interest.

## Appendix

**TABLE A1:** Romantic and non-romantic words used in the experiment.

**Romantic words**					
动词	Verb	名词	Noun	形容词	Adjective
思念	Miss	婚礼	Wedding	深情	Soulful
求婚	Propose	伴侣	Mate	芳香	Fragrant
结婚	Marry	爱人	Spouse	喜欢	Fond
祝福	Bless	爱情	Affection	永恒	Everlasting
约会	Date	戒指	Ring	亲密	Close
恋爱	Love	情侣	Lovers	亲爱	Dear
爱慕	Adore	诺言	Promise	亲昵	Intimacy
接吻	Kiss	玫瑰	Rose	痴心	Spoony
**Non-romantic words**					
动词	Verb	名词	Noun	形容词	Adjective
录取	Admit	财富	Wealth	崇高	Lofty
奖励	Reward	奖金	Bonus	年轻	Young
提拔	Promote	冠军	Champion	诚挚	Sincere
荣获	Award	友谊	Friendship	吉利	Lucky
欢呼	Cheer	盈利	Profit	精彩	Splendid
表扬	Praise	笑话	Joke	热火	Passionate
表彰	Commend	正义	Justice	机智	Tactful
取胜	Win	创新	Innovate	尊重	Respectful

## References

[B1] Ackerman J. M. Nocera C. C. Bargh J. A. (2010). Incidental haptic sensations influence social judgments and decisions. Science 328, 1712–1715. 10.1126/science.1189993 20576894 PMC3005631

[B2] Amsel B. D. Cree G. S. (2013). Semantic richness, concreteness, and object domain: an electrophysiological study. Can. J. Exp. Psychol. 67, 117–129. 10.1037/a0029807 23046416

[B3] Barsalou L. W. (2003). Situated simulation in the human conceptual system. Lang. Cogn. Process. 18, 513–562. 10.1080/01690960344000026

[B4] Barsalou L. W. (2008). Grounded cognition. Annu. Rev. Psychol. 59, 617–645. 10.1146/annurev.psych.59.103006.093639 17705682

[B5] Bartels A. Zeki S. (2000). The neural basis of romantic love. Neuroreport 11, 3829–3834. 10.1097/00001756-200011270-00046 11117499

[B6] Bernat E. Bunce S. Shevrin H. (2001). Event-related brain potentials differentiate positive and negative mood adjectives during both supraliminal and subliminal visual processing. Int. J. Psychophysiol. 42, 11–34. 10.1016/S0167-8760(01)00133-7 11451477

[B7] Chan K. Q. Tong E. M. Tan D. H. Koh A. H. (2013). What do love and jealousy taste like? Emotion 13, 1142–1149. 10.1037/a0033758 24040883

[B8] Chandler J. J. Reinhard D. Schwarz N. (2012). To judge a book by its weight you need to know its content: knowledge moderates the use of embodied cues. J. Exp. Soc. Psychol. 48, 948–952. 10.1016/j.jesp.2012.03.003

[B9] Denke C. Rotte M. Heinze H. J. Schaefer M. (2016). Lying and the subsequent desire for toothpaste: activity in the somatosensory cortex predicts embodiment of the moral-purity metaphor. Cereb. Cortex 26, 477–484. 10.1093/cercor/bhu170 25214311

[B10] Dong G. Hu Y. Zhou H. (2010). Event-related potential measures of the intending process: time course and related ERP components. Behav. Brain Funct. 6:15. 10.1186/1744-9081-6-15 20178644 PMC2848189

[B11] Förster J. Liberman N. (2007). Knowledge activation, in Social Psychology: Handbook of Basic Principles, 2nd Edn. eds Kruglanski A. W. Higgins I. E. T. (New York, NY: Guilford Press), 201–231.

[B12] Grondin R. Lupker S. J. McRae K. (2009). Shared features dominate semantic richness effects for concrete concepts. J. Mem. Lang. 60, 1–19. 10.1016/j.jml.2008.09.001 20046224 PMC2634287

[B13] Hauser D. J. Schwarz N. (2015). Elaborative thinking increases the impact of physical weight on importance judgments. Soc. Cogn. 33, 120–132. 10.1521/soco.2015.33.2.120

[B14] Higgins E. T. (1996). Knowledge activation: accessibility, applicability, and salience, in Social Psychology: Handbook of Basic Principles, eds Higgins I. E. T. Kruglanski A. W. (New York, NY: Guilford Press, 133–168.

[B15] Kanske P. Kotz S. A. (2007). Concreteness in emotional words: ERP evidence from a hemifield study. Brain Res. 1148, 138–148. 10.1016/j.brainres.2007.02.044 17391654

[B16] Kounios J. Green D. L. Payne L. Fleck J. I. Grondin R. Mcrae K. (2009). Semantic richness and the activation of concepts in semantic memory: evidence from event-related potentials. Brain Res. 1282, 95–102. 10.1016/j.brainres.2009.05.092 19505451 PMC2709703

[B17] Kwon Y. Nam K. Lee Y. (2012). ERP index of the morphological family size effect during word recognition. Neuropsychologia 50, 3385–3391. 10.1016/j.neuropsychologia.2012.09.041 23036281

[B18] Landau M. J. Vess M. Arndt J. Rothschild Z. K. Sullivan D. Atchley R. A. (2011). Embodied metaphor and the “true” self: priming entity expansion and protection influences intrinsic self-expressions in self-perceptions and interpersonal behavior. J. Exp. Soc. Psychol. 47, 79–87. 10.1016/j.jesp.2010.08.012

[B19] Lau E. F. Phillips C. Poeppel D. (2008). A cortical network for semantics:(de) constructing the N400. Nat. Rev. Neurosci. 9, 920–933. 10.1038/nrn2532 19020511

[B20] Lee S. W. Schwarz N. (2010). Dirty hands and dirty mouths embodiment of the moral-purity metaphor is specific to the motor modality involved in moral transgression. Psychol. Sci. 21, 1423–1425. 10.1177/0956797610382788 20817782

[B21] Lee S. W. Schwarz N. (2014). Framing love: when it hurts to think we were made for each other. J. Exp. Soc. Psychol. 54, 61–67. 10.1016/j.jesp.2014.04.007

[B22] Lee S. W. S. Schwarz N. (2011). Wiping the slate clean psychological consequences of physical cleansing. Curr. Dir. Psychol. Sci. 20, 307–311. 10.1177/0963721411422694

[B23] Lee S. W. S. Schwarz N. (2012). Bidirectionality, mediation, and moderation of metaphorical effects: the embodiment of social suspicion and fishy smells. J. Pers. Soc. Psychol. 103, 737–749. 10.1037/a0029708 22905770

[B24] Levy-Drori S. Henik A. (2006). Concreteness and context availability in lexical decision tasks. Am. J. Psychol. 119, 45–65. 10.2307/20445318 16550855

[B25] Li J. Wang L. (2018). Sweetness enhancement effect of romantic semantics (in Chinese). Psychol. Tech. Appl. 6, 746–751. 10.16842/J.cnki.issn2095-5588.2018.12.001

[B26] Luo S. Han X. Du N. Han S. (2018). Physical coldness enhances racial in-group bias in empathy: electrophysiological evidence. Neuropsychologia 116, 117–125. 10.1016/j.neuropsychologia.2017.05.002 28478242

[B27] Pecher D. Zeelenberg R. Barsalou L. W. (2003). Verifying conceptual properties in different modalities produces switching costs. Psychol. Sci. 14, 119–124. 10.1111/1467-9280.t01-1-01429 12661672

[B28] Pexman P. M. Hargreaves I. S. Edwards J. D. Henry L. C. Goodyear B. G. (2007). The neural consequences of semantic richness: when more comes to mind, less activation is observed. Psychol. Sci. 18, 401–406. 10.1111/j.1467-9280.2007.01913.x 17576279

[B29] Pexman P. M. Hargreaves I. S. Siakaluk P. Bodner G. Pope J. (2008). There are many ways to be rich: effects of three measures of semantic richness on word recognition. Psychon. Bull. Rev. 15, 161–167. 10.3758/PBR.15.1.161 18605497

[B30] Rabovsky M. Sommer W. Abdel Rahman R. (2012). The time course of semantic richness effects in visual word recognition. Front. Hum. Neurosci. 6:11. 10.3389/fnhum.2012.00011 22347855 PMC3278705

[B31] Razumiejczyk E. Macbeth G. Marmolejo-Ramos F. Noguchi K. (2015). Crossmodal integration between visual linguistic information and flavour perception. Appetite 91, 76–82. 10.1016/j.appet.2015.03.035 25843936

[B32] Razumiejczyk E. Pereyra Girardi C. Crivello M. D. C. Fioramonti M. Macbeth G. Marmolejo-Ramos F. (2017). Crossmodal interference between language and flavour. Rev. Latinoam. Psicol. 49, 91–101. 10.1016/j.rlp.2016.01.002

[B33] Ren D. Tan K. Arriaga X. B. Chan K. Q. (2014). Sweet love: the effects of sweet taste experience on romantic perceptions. J. Soc. Pers. Relat. 32, 905–921. 10.1177/0265407514554512

[B34] Schwanenflugel P. J. Shoben E. J. (1983). Differential context effects in the comprehension of abstract and concrete verbal materials. J. Exp. Psychol. Learn. Mem. Cogn. 9, 82–102. 10.1037/0278-7393.9.1.82

[B35] Simmons W. K. Ramjee V. Beauchamp M. S. McRae K. Martin A. Barsalou L. W. (2007). A common neural substrate for perceiving and knowing about color. Neuropsychologia 45, 2802–2810. 10.1016/j.neuropsychologia.2007.05.002 17575989 PMC3596878

[B36] Spence C. Deroy O. (2013). Crossmodal mental imagery, in Multisensory Imagery, eds Lacey S. Lawson R. (New York, NY: Springer, 157–183.

[B37] Spence C. Nicholls M. E. R. Driver J. (2001). The cost of expecting events in the wrong sensory modality. Percept. Psychophys. 63, 330–336. 10.3758/BF03194473 11281107

[B38] Tillman R. Hutchinson S. Jordan S. Louwerse M. (2013). Verifying properties from different emotions produces switching costs: evidence for coarse-grained language statistics and fine-grained perceptual simulation, in Proceedings of the Annual Meeting of the Cognitive Science Society, Vol. 35 Available online at: https://escholarship.org/uc/item/69w9r6fc

[B39] Todorov A. (2000). The accessibility and applicability of knowledge: predicting context effects in national surveys. Public Opin. Q. 64, 429–451. 10.1086/318639 11171025

[B40] Tousignant C. Pexman P. M. (2012). Flexible recruitment of semantic richness: context modulates body–object interaction effects in lexical-semantic processing. Front. Hum. Neurosci. 6:53. 10.3389/fnhum.2012.00053 22435058 PMC3304254

[B41] Van Dantzig S. Pecher D. Zeelenberg R. Barsalou L. W. (2008). Perceptual processing affects conceptual processing. Cogn. Sci. 32, 579–590. 10.1080/03640210802035365 21635347

[B42] Velasco C. Beh E. Le T. Marmolejo-Ramos F. (2018). The shapes associated with the concept of ‘sweet and sour’ foods. Food Qual. Prefer. 68, 250–257. 10.1016/j.foodqual.2018.03.012

[B43] Vermeulen N. Niedenthal P. M. Luminet O. (2007). Switching between sensory and affective systems incurs processing costs. Cogn. Sci. 31, 183–192. 10.1080/03640210709336990 21635293

[B44] Wang H. Mo L. Luo Q. Xiang Y. Hu Y. (2015). Psychological distance modulates the performance of the embodiment effect: evidence from behavioral and ERP studies. Psychophysiology 53, 527–534. 10.1111/psyp.12577 26589656

[B45] Wang L. Chen Q. (2018). Experiencing sweet taste affects romantic semantic processing. Curr. Psychol. 37, 1–9. 10.1007/s12144-018-9877-8

[B46] Wang L. Li J. (2014). The switching effect involving the affective system in chinese affective concept processing. Univ. J. Psychol. 2, 151–157. 10.13189/ujp.2014.020501

[B47] Wang L. Liu T. Chen Y. Zhong R. Zhang H. Dai M. (2019). The concreteness effect of word processing for highly neurotic individuals: an event-related potential study. Neuroreport 30, 305–309. 10.1097/WNR.0000000000001201 30688758

[B48] Wang L. Yao W. (2016). So Near, so good: does near-distance perception reduce interpersonal psychological distance? Soc. Behav. Pers. Int. J. 44, 889–898. 10.2224/sbp.2016.44.6.889

[B49] Wang Y. N. Zhou L. M. Luo Y. J. (2008). The pilot establishment and evaluation of Chinese affective words system[In Chinese]. Chin. Ment. Health J. 22, 608–612. 10.3321/j.issn:1000-6729.2008.08.014

[B50] West W. C. Holcomb P. J. (2006). Imaginal, semantic, and surface-level processing of concrete and abstract words: an electrophysiological investigation. J. Cogn. Neurosci. 12, 1024–1037. 10.1162/08989290051137558 11177422

[B51] Williams L. E. Bargh J. A. (2008). Experiencing physical warmth promotes interpersonal warmth. Science 322, 606–607. 10.1126/science.1162548 18948544 PMC2737341

[B52] Wyer R. S. (2008). The role of knowledge accessibility in cognition and behavior. Handb. Consum. Psychol. 4, 31–76. 10.4324/9780203809570.ch2

[B53] Xue J. Liu T. Marmolejo-Ramos F. Pei X. (2017). Age acquisition effects on word processing for Chinese native learner's English: ERP evidence for the arbitrary mapping hypothesis. Front. Psychol. 8:818 10.3389/fpsyg.2017.00818 28572785 PMC5435808

[B54] Yap M. J. Pexman P. M. Wellsby M. Hargreaves I. S. Huff M. J. (2012). An abundance of riches: cross-task comparisons of semantic richness effects in visual word recognition. Front. Hum. Neurosci. 6:72. 10.3389/fnhum.2012.00072 22529787 PMC3328122

[B55] Zanolie K. Dantzig S. V. Boot I. Wijnen J. Schubert T. Giessner S.. (2012). Mighty metaphors: behavioral and ERP evidence that power shifts attention on a vertical dimension. Brain Cogn. 78, 50–58. 10.1016/j.bandc.2011.10.006 22088775

